# Compromising the Unfolded Protein Response Induces Autophagy-Mediated Cell Death in Multiple Myeloma Cells

**DOI:** 10.1371/journal.pone.0025820

**Published:** 2011-10-18

**Authors:** Anne-Sophie Michallet, Paul Mondiere, Morgan Taillardet, Yann Leverrier, Laurent Genestier, Thierry Defrance

**Affiliations:** 1 Institut National de la Santé et de la Recherche Médicale, Lyon, France; 2 Université de Lyon, Biosciences Lyon-Gerland, Lyon, France; 3 Hospices Civils de Lyon, Centre Hospitalier Lyon Sud, Pierre Bénite, France; Yale Medical School, United States of America

## Abstract

**Objective:**

To determine whether the Unfolded Protein Response (UPR) sensors (PERK, ATF6 and IRE-1) can be targeted to promote death of Multiple Myeloma (MM) cells.

**Methods:**

We have knocked-down separately each UPR stress sensor in human MM cell lines using RNA interference and followed MM cell death by monitoring the membrane, mitochondrial and nuclear alterations. Involvement of caspases in MM cell death consecutive to UPR sensor knock-down was analyzed by western blotting, measurement of their enzymatic activity using fluorigenic substrates and susceptibility to a pan-caspase inhibitor. Activation of the autophagic process was measured directly by detection of autophagosomes (electronic microscopy), monodansylcadaverine staining, production of the cleaved form of the microtubule-associated protein 1A/1B light chain 3 (LC3) and indirectly by analyzing the impact of pharmacological inhibitors of autophagy such as 3MA and bafilomycin A1.

**Results:**

We show that extinction of a single UPR stress sensor (PERK) induces a non-apoptotic form of cell death in MM cells that requires autophagy for its execution. We also show that this cytotoxic autophagic process represses the apoptosis program by reducing the cytosolic release of the apoptogenic factors Smac/DIABLO and cytochrome c.

**Interpretation:**

Altogether our findings suggest that autophagy can contribute to execution of death in mammalian cells that are exposed to mild ER stress. They also suggest that the autophagic process can regulate the intrinsic apoptotic pathway by inhibiting production of death effectors by the mitochondria, thus preventing formation of a functional apoptosome. Altogether these findings give credit to the idea that UPR sensors can be envisaged as therapeutic targets for the treatment of MM.

## Introduction

Multiple Myeloma (MM) is a plasma cell (PC) malignancy mainly localized in the bone marrow and characterized by the secretion of high levels of paraprotein in the serum and/or urine. Despite the progress made in chemotherapy and stem cell transplantation, the development of promising options such as antiangiogenic drugs or proteasome inhibitors [1], [2], MM remains an incurable disease. Both normal and tumoral PC have expanded their Endoplasmic Reticulum (ER) to accommodate high-rate Ig synthesis [3]. Changes in the ER that interfere with the proper maturation of secreted proteins initiate a coordinated adaptive program called the UPR [4], [5], [6], [7]. The UPR is a complex multimolecular machinery that senses a variety of ER stress conditions and triggers multiple signaling pathways that cooperate to alleviate ER stress [8], [9], [10]. UPR induction results in an initial decrease in general protein synthesis that reduces the influx of nascent proteins into the ER. It also increases transcription of ER resident chaperones, folding enzymes and components of the protein degradative machinery to prevent aggregation of the accumulating misfolded proteins. This concerted and complex cellular response is mediated through three ER transmembrane receptors: pancreatic ER kinase (PKR)-like ER kinase (PERK) [11], [12], [13], [14], activating transcription factor 6 (ATF6) [15], [16], [17] and inositol-requiring enzyme 1 (IRE-1) [18], [19], [20]. These three ER proteins act as «stress sensors» and are the most proximal inducers of the UPR.

The «physiological» or cytoprotective UPR allows cells to survive a transient overload of the ER [21]. If the stress is excessive or prolonged and homeostasis cannot be restored, the UPR initiates cell death, a phase designated as terminal or cytotoxic UPR. Accumulating data indicate that ER stress is also a potent trigger of autophagy [22]. Autophagy is a highly conserved process that intervenes to maintain cellular integrity in response to stress. It allows for elimination of damaged organelles and recycling of this cellular material to provide energy and building blocks that help cells to survive nutrient or growth factor deprivation [23]. The question as to whether autophagy can also cause cell death is controversial [24], [25]. Our previous work suggested that the highly developed secretory apparatus of PC may also sensitize them to ER stress-induced apoptosis [26]. Furthermore, Multiple Myeloma cell lines constitutively express high levels of UPR components and the PERK pathway has been shown to be constitutively active in these cells [11]. We reasoned that MM cells may heavily rely on the UPR for their survival which led us to explore the possibility to target ER stress sensors in order to trigger their death. Here, we show that knock-down of a single UPR sensor leads to the autophagic cell death of human MM cell lines. We bring evidence that autophagy represses the apoptosis program by instructing mitochondria to limit the release of the apoptogenic factors and the subsequent activation of caspases. Our findings provide additional evidence that autophagy can contribute to execution of death in mammalian cells that have been exposed to a mild ER stress. They also suggest that intensity of the ER stress signal and of the subsequent UPR does not only control the survival/death decision but also the choice of the death modality.

## Results

### Extinction of the UPR sensors induces death of human MM cell lines

**Figure 1:**
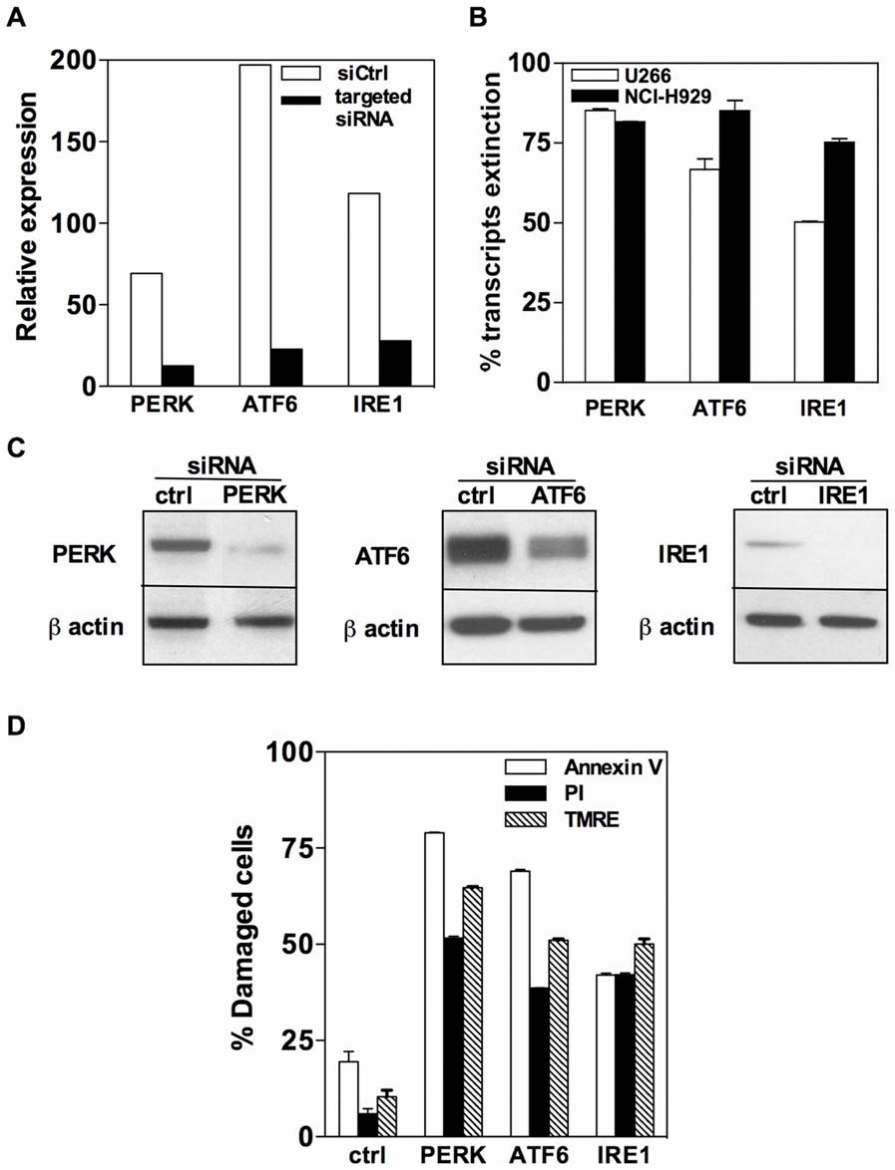
Silencing UPR sensors induces death of human MM cell lines. A. NCI-H929 cells were transfected with the PERK, ATF6, IRE1 or non-targeting siRNAs. The levels of expression of the targeted transcripts were determined by real-time RT-PCR 24 h after transfection. B. Percentages of transcript extinction induced by the targeting siRNAs in U266 and NCI-H929 cells (mean ± SD values of three independent experiments). C. Whole cell lysates were prepared from NCI-H929 cells transfected with the targeting or non-targeting siRNAs and blotted with anti-PERK, anti-ATF6, anti IRE1 or anti-ß actin mAbs. Representative of two independent experiments. D. NCI-H929 cells were transfected with the PERK (siPERK) or control siRNA (siCtrl). Percentages of cells with Δm loss (TMRE^lo^) or with membrane alterations (Annexin V+ or PI+) were evaluated by TMRE and annexin V or PI stainings, respectively. Results represent the mean+SD values of 2 experiments.

As shown in Figure 1 (A and B), transient transfection of siRNAs against PERK, ATF6 and IRE1 strongly down-regulated expression of their respective target transcripts in both the U266 and NCI-H929 MM cell lines and reduced expression of the corresponding proteins (Figure 1C). PS exposure (via annexin V staining) and propidium iodide (PI) exclusion, that both monitor plasma membrane alterations, as well as TMRE staining that reveals disruption of the mitochodrondrial transmembrane potential, were used to estimate the death rate of MM cells transfected with siRNAs targeting UPR sensors. As illustrated by Figure 1D, all three UPR sensor-targeted siRNAs induced both membrane and mitochondrial alerations as compared to the control siRNA. The PERK siRNA was reproducibly found to be the most efficient death inducer. The death rate induced by siRNAs targeting UPR sensors was maximal 24 hours after transfection (not shown). Altogether, our findings indicate that transient extinction of the UPR sensors, promotes death of human MM cells.

### Knock-down of a single UPR sensor amplifies the UPR in MM cells

**Figure 2:**
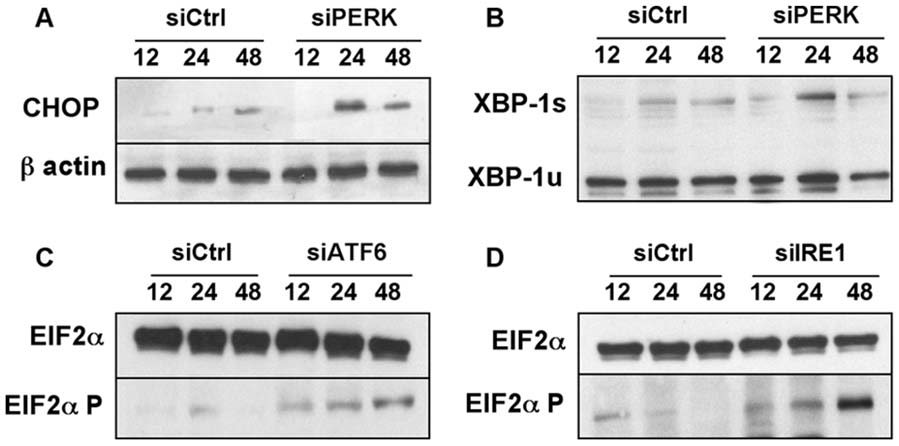
Extinction of the UPR sensors induces transient activation of the UPR in MM cells. The NCI-H929 MM cell line was transfected with the PERK (A, B), ATF6 (C), IRE1 (D) or non-targeting (ctrl) siRNAs. Whole cell lysates were prepared 12, 24 or 48 h after transfection and analyzed by Western blot for the expression of CHOP, unspliced XBP-1 (u), spliced XBP-1 (s), total and phosphorylated EIF2α and β actin. The data are representative of three different experiments.

Because PERK−/− tissues are characterized by an enhanced activation of the PERK- independent branches of the UPR [13], we postulated that knocking down one UPR sensor could amplify signaling through the other two ER stress pathways. To address this question, we monitored the impact of the three UPR sensor-targeted siRNAs on expression of downstream effectors of the UPR. Production of the phosphorylated form of eIF-2 and of the spliced form of XBP-1 were used to reveal activation of the PERK and IRE1 pathways respectively. Expression of the GADD153/CHOP transcription factor was used as a general indicator of UPR activation. As illustrated by Figure 2, low levels of CHOP, cleaved XBP-1 and the phosphorylated form of eIF-2 alpha, were detected in the MM cell line U266 transfected with the control siRNA. Transfection of the PERK siRNA (Figure 2, A and B) induced a transient upregulation of CHOP and of the spliced form of XBP-1 that both peaked at 24 hours. Similarly, siRNA-induced knock-down of ATF6 or IRE1 (Figure 2, C and D) enhanced expression of the phosphorylated form of eIF-2 alpha, signing activation of the PERK pathway. These data suggest that knock-down of a single UPR sensor exacerbates the signals transduced by the other two intact sensors thus resulting in a transient burst of UPR activity.

### MM cell death promoted by UPR sensor knock-down is distinct from apoptosis

**Figure 3:**
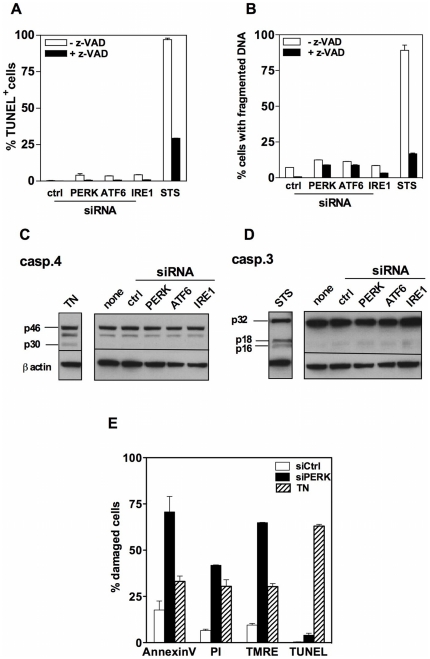
Silencing UPR sensors fails to promote DNA fragmentation and caspase activation in MM cells. A–B. NCI-H929 cells were transfected with the targeting or non-targeting siRNAs and cultured for 24 h with or without zVAD-fmk (100 µM). Cells were also treated with staurosporine (STS) as a positive control for caspase-dependent cell death. Oligonucleosomal DNA fragmentation was estimated both by the TUNEL (A) and the Apostain (B) assays. Results are expressed as the mean ± SD percentages of cells with fragmented DNA as calculated from duplicate determinations. The data shown are representative of three independent experiments. C–D. Whole cell extracts were prepared from NCI-H929 cells transfected either with the targeting or the non targeting siRNA or treated with tunicamycin (TN) or STS for 24 h. Activation of caspase-4 (C) and caspase-3 (D) was assessed by immunoblot analysis. The anti-ß actin mAb was used as a loading control. The proenzymatic forms of caspase-4 and caspase-3 were visualized as a 46 kD and a 32 kD band, respectively. Their cleaved active fragments were identified as a 30 kD band (caspase-4) and as a 16–18 kD doublet (caspase-3) (representative of three independent experiments). E. NCI-H929 cells were transfected with the non-targeting or PERK siRNAs or treated with tunicamycin (TN) for 24 h and examined for membrane, mitochondrial and nuclear alterations. Results are expressed as the mean ± SD percentages of positive cells (for annexin V, PI and TUNEL) or TMRE^low^ cells as calculated from duplicate determinations.

To define how MM cell death induced through UPR sensor silencing relates to apoptosis we analyzed the impact of UPR sensor-targeted siRNA on oligonucleosomal DNA fragmentation and activation of caspases, which are key players of apoptotic cell death. In contrast to staurosporine that gave a strong signal in both the TUNEL and Apostain assays, the UPR sensor-targeted siRNAs failed to promote DNA fragmentation (Figure 3, A and B). The staurosporine-induced DNA fragmentation in MM cell lines was efficiently repressed by the pan-caspase inhibitor zVAD-fmk thus confirming that caspases are involved in the process. The lack of DNA fragmentation in response to UPR sensor knock-down strongly suggested that caspases do not contribute to MM cell death in this context. However, caspases may also be activated without directly contributing to cell death. To test this possibility, we monitored the impact of UPR sensor-targeted siRNAs on the cleavage of the ER-associated initiator caspase 4 and of the effector caspase-3. As shown in Figure 3, C and D, neither caspase-4 nor caspase-3 were activated by target siRNAs in NCI-H929 MM cells while pharmacological death inducers promoted cleavage of caspase-4 (tunicamycine) and caspase-3 (staurosporine) in the same cells. In contrast with UPR sensor-targeted siRNAs, pharmacological ER stressors such as tunicamycin have been reported to promote apoptosis of MM cells [27]. We thus determined whether the non-apoptotic death triggered by UPR sensor-targeting siRNAs is a specificity of this peculiar ER stress stimulus or a singularity of the response of our MM cell lines to ER stress. For this purpose, we compared the PERK siRNA and tunicamycin for their ability to induce membrane, mitochondrial and nuclear alterations in NCI-H929 cells. As opposed to the PERK siRNA, tunicamycin not only caused membrane and mitochondrial alterations but also gave a strong signal in the TUNEL assay thereby demonstrating that NCI-H929 cells are fully apoptosis-competent in response to pharmacological ER stressors (Figure 3E). Altogether, these findings suggest that, as opposed to pharmacological ER stress inducers, extinction of a single UPR sensor in MM promotes a caspase-independent non-apoptotic form of programmed cell death.

### Silencing UPR sensors induces autophagic cell death of MM cells

**Figure 4:**
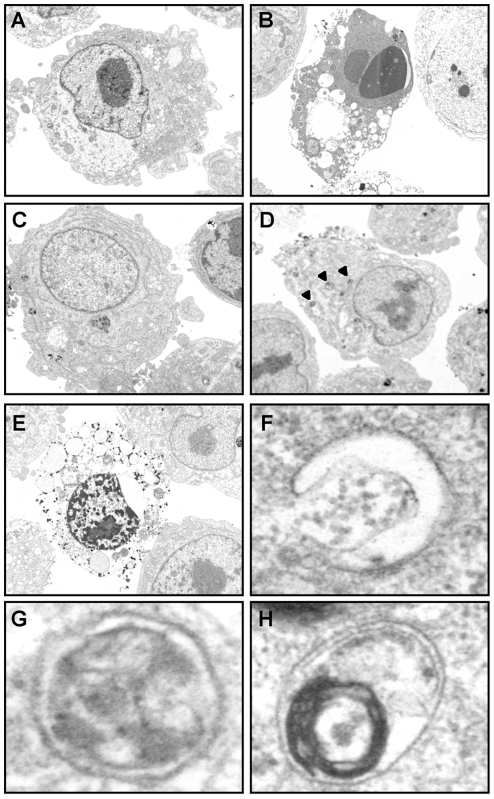
The cell death promoted by the PERK siRNA is morphologically distinct from apoptosis. NCI-H929 cells were either transfected with the non-targeting (C) or with the PERK (D–H) siRNA, treated with staurosporine (B) or left untreated (A) for 24 h and then examined by electron microscopy. The two stages or the death process induced by PERK silencing are illustrated in panels D and E. F–H Visualization at higher magnification of the cytosolic electron-dense structures identified by the arrowheads in D. In F, a pre-autophagosomal-like structure, in G and H, autophagosomes with a double membrane sequestering cellular material.

**Figure 5:**
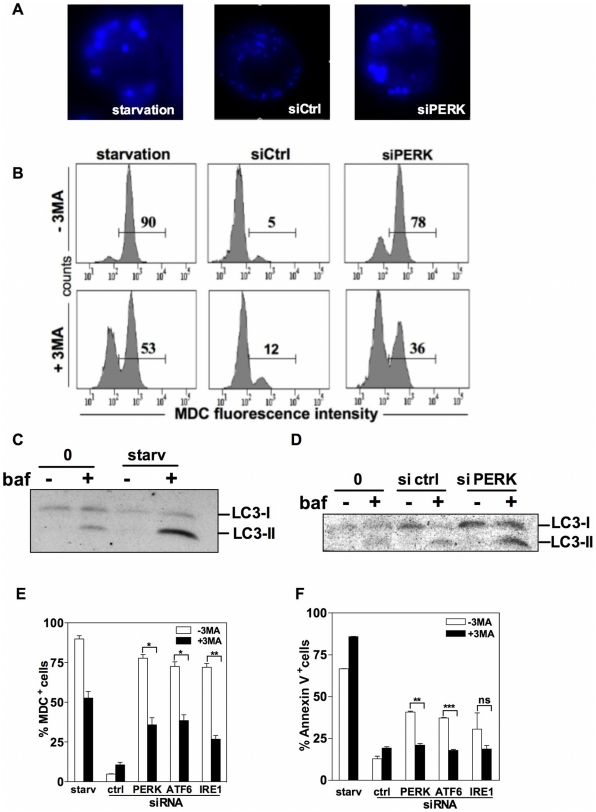
Extinction of the UPR sensors induces the autophagic cell death of MM cells. A. NCI-H929 cells were serum-starved or transfected with the non-targeting or PERK siRNAs for 24 h then stained with MDC and examined by fluorescence microscopy. B. NCI-H929 cells cultured as in A, in the presence or absence of 3-MA, were stained with MDC and analyzed by flow cytometry. C and D, NCI-H929 cells were serum-starved (C) or transfected either with the non-targeting or the PERK siRNAs (D). All cultures were conducted with or without bafilomycin A1. Accumulation of autophagosomes was visualized by the conversion of endogenous LC3-I (18 kDa) to LC3-II (16 kDa). E. Average proportion of MDC^+^ cells in NCI-H929 cells 24 h after transfection with the targeting or non-targeting siRNAs or 24 h after culture in serum-starved conditions. All cultures were conducted with or without 3-MA. Results are expressed as the mean ± SD percentages of positive cells as calculated from duplicate determinations. The data shown are representative of two independent experiments. F. NCI-H929 cells were cultured as in E. Membrane alterations were estimated by Annexin V staining after 24 h of culture. Results are expressed as the mean ± SD percentages of positive cells as calculated from duplicate determinations and are representative of three independent experiments. (* *p*<0.05; ** *p*<0.01; ***p<0.005; *ns* = non significant).

To define the type of non-apoptotic cell death triggered by extinction of the UPR sensors in MM cells, we first examined the morphology of NCI-H929 cells after exposure to different death-inducers by transmission electron microscopy. As shown in Figure 4, while cells treated with STS presented a typical apoptotic morphology with chromatin condensation and nuclear fragmentation (Figure 4B), transfection with the PERK siRNA generated two types of cells with altered morphology (Figures 4D and E) that we designed as stage I and II cells. Stage I cells have an apparently normal nuclear morphology but present numerous electron dense inclusions in the cytosol (Figure 4D). Upon magnification, these inclusions appeared to be double-layered membrane stuctures evocative of autophagosomes (Figures 4 G and H) or crescent-like structures ressembling the early stage of autophagosome formation (Figure 4F). Stage II cells are characterized by partial chromatin condensation and extensive vacuolization of the cytoplasm (Figure 4E). To confirm that autophagy occurs during this process, NCI-H929 cells were stained with MDC, a fluorescent dye that is selectively incorporated into autophagosomes and autolysosomes. As shown in Figure 5A, serum starvation, a physiological stimulus of autophagy, increased the number and size of the MDC-labeled vesicles in MM cells. The PERK siRNA but not the control siRNA also induced coalescence of the MDC-labeled vesicles, a result which was confirmed by the flow cytometry analysis of MDC incorporation in MM cells (Figure 5B). Furthermore, the pharmacological autophagy blocker 3-MA significantly reduced the MDC staining both in starved cells and in cells transfected with the UPR-targeted siRNAs (Figure 5, B and E). To provide further evidence of the implication of the autophagic process in the demise of MM cells transfected with the PERK siRNA, experiments were then conducted with bafilomycin A1 that blocks the fusion of autophagosomes with lysosomes. In cells, which are exposed to an active autophagic process, bafilomycin A1 causes accumulation of autophagosomes that can be visualized by the conversion of endogeneous LC3-I to LC3-II. Bafilomycin A1 also revealed enhanced production of the cleaved LC3 form in cells cultured in serum-starved conditions and in cells transfected with PERK siRNA as compared with control cultures, i.e. untreated cells and cells transfected with the non targeting siRNA, respectively (Figure 5, C and D). These data indicate that MM cell death induced by extinction of a single UPR sensor is either accompanied or caused by autophagy. To determine whether the autophagic process initiated by UPR sensor knock-down is cytoprotective or cytotoxic, we first analyzed the impact of 3-MA on PS exposure. The proportions of annexin V^+^ cells was enhanced by 3-MA in serum-starved cultures but reduced by half in cells transfected by UPR-targeted siRNAs (Figure 5F). Altogether our findings suggest that extinction of the UPR sensors initiates a cytotoxic autophagic process.

### Autophagy induced by UPR sensor extinction represses apoptosis of MM cells

**Figure 6:**
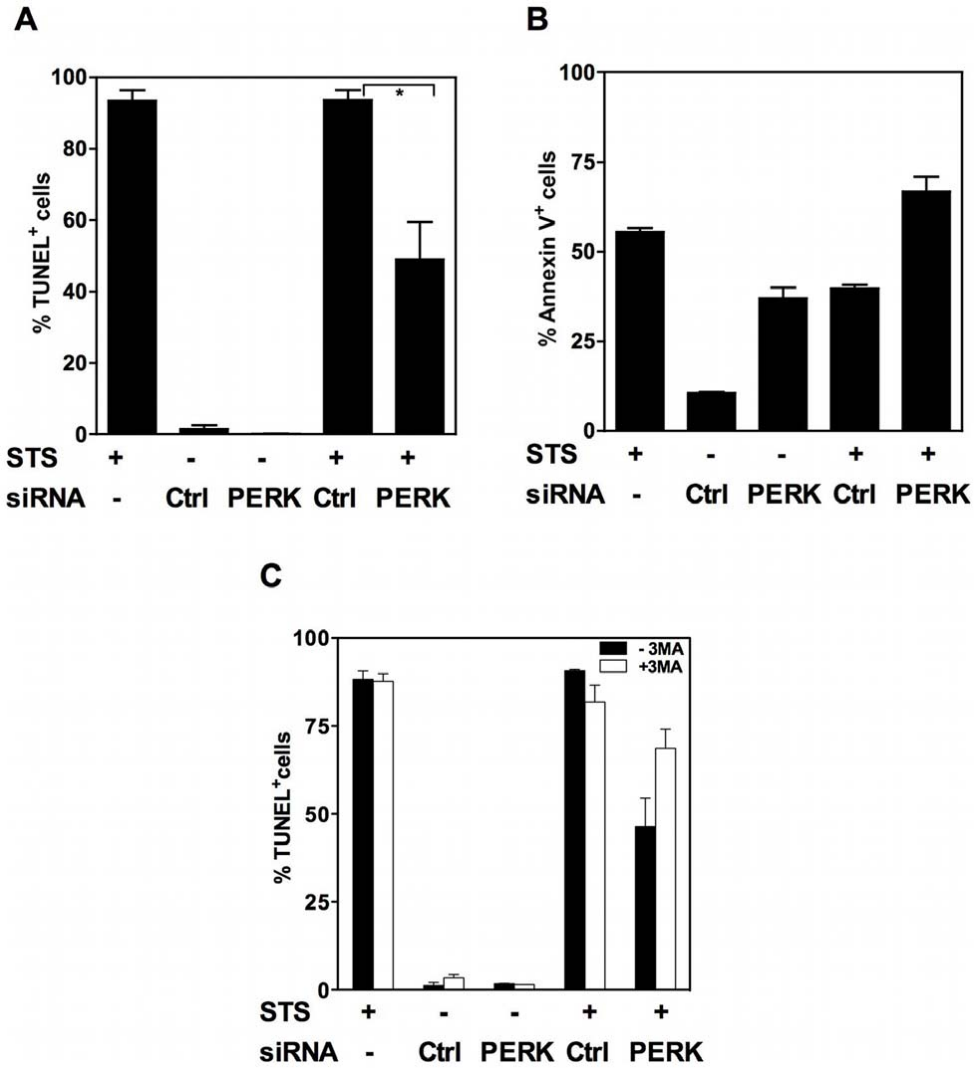
PERK silencing represses apoptosis of MM cells. A. NCI-H929 cells were transfected with the non-targeting or PERK siRNA and treated or not with STS 8 h later. Control cultures in which untransfected cells were treated with STS were also conducted. DNA fragmentation (A) and PS exposure (B) were assessed 24 h after transfection. Results are expressed as the mean ± SD percentages of positive cells as calculated from duplicate determinations and are representative of two independent experiments. C. NCI-H929 cells were cultured as in B and C in the presence or absence of 3-MA. DNA fragmentation was assessed by the TUNEL assay. Mean ± SD values of two independent experiments are shown. (* *p*<0.05).

We next explored the possibility that knock-down of a single UPR sensor could induce blockade of the apoptosis pathway. For this purpose, NCI-H929 cells were treated with an apoptosis-inducing stimulus (staurosporine) 8 hours after transfection with the PERK or control siRNAs. Both membrane (annexin V staining) and nuclear (TUNEL assay) alterations were monitored at the end of the culture. As illustrated by Figure 6, A and B, the PERK siRNA, but not the control siRNA, significantly reduced the DNA fragmentation levels induced by staurosporine while it cooperated with staurosporine for induction of PS exposure. This observation suggests that the PERK siRNA actively represses induction of apoptosis. We next addressed the question whether repression of apoptosis triggered by PERK silencing was or not consecutive to the induction of autophagy. For this purpose NCI-H929 cells were transfected or not with either the PERK or the control siRNA before being exposed to staurosporine, in the presence or absence of 3-MA. Cells were then processed for analysis of DNA fragmentation. As illustrated by Figure 6C, addition of 3-MA did not alter the levels of DNA fragmentation induced by staurosporine or the PERK siRNA but partially restored the rates of staurosporine-induced DNA fragmentation in cultures co-treated with staurosporine and the PERK siRNA. Altogether, these findings suggest that the autophagic process represses the apoptosis program in our experimental model.

### UPR sensor silencing impairs the mitochondrial apoptogenic potential

**Figure 7:**
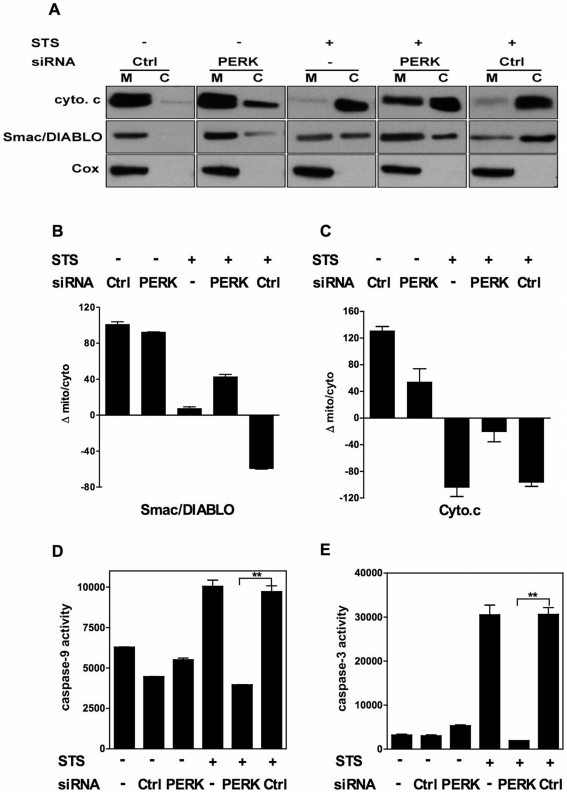
UPR sensor silencing impairs the apoptogenic potential of the mitochondria. A. NCI-H929 cells were transfected or not with the non-targeting or PERK siRNAs before being treated or not with STS. Cytosolic and mitochondrial protein extracts were prepared 24 h after transfection and immunobloted with Abs directed against: Smac/DIABLO, cytochrome c (cyt. c) or cytochrome oxidase (Cox). B, C. A Java-based image processing (Image J) was used to estimate the densitometric intensity of the bands on the immunoblot. Results for cytochrome c and Smac/DIABLO are presented in B and C, respectively. D, E. NCI-H929 cells were transfected with the non-targeting or the PERK siRNA and treated or not with STS. Caspase-9 (D) and caspase-3 (E) activities were measured 24 h after transfection. Results are expressed as the mean ± SD absorbance values as calculated from duplicate determinations and are representative of two independent experiments. (** *p*<0.01).

Mitochondria play a crucial role in initiation of the intrinsic apoptosis pathway by releasing, after permeabilization of their outer membrane, apoptogenic factors such as cytochrome c, apoptosis-inducing factor (AIF) or Smac/DIABLO. When released in the cytosol, cytochrome c binds the cytosolic molecule Apaf-1 to initiate formation of the apoptosome. Once formed, the apoptosome can then recruit and activate the inactive pro-caspase-9. Once activated, this initiator caspase can then activate effector caspases such as caspase-3 and trigger a cascade of events leading to apoptosis. AIF causes large-scale DNA fragmentation and Smac neutralizes the inhibitor of apoptosis proteins (IAPs) that repress caspase activation. To estimate whether extinction of the UPR sensor alter the apoptosis effector function of the mitochondria, NCI-H929 cells transfected or not with either the PERK or the control siRNA were subsequently exposed or not to staurosporine. Cytosolic and mitochondrial subcellular fractions were prepared 24 h after transfection and the protein extracts were immunobloted with anti-cytochrome oxidase (Cox), anti-cytochrome c and anti-Smac/DIABLO Abs. To compare the amounts of cytosolic cytochrome c and Smac/DIABLO release in different culture conditions, a densitometry scanning of the blots was performed and we calculated for each band the Δ mito/cyto value by subtracting the staining intensity of the cytosolic fraction from that of the mitochondrial fraction. As shown in Figure 7A, Cox was exclusively found in the mitochondrial fraction in all culture conditions thus testifying that mitochondria integrity was preserved during the subcellular fractionation procedure. STS induced a nearly complete loss of cytochrome c from the mitochondria and relocation of approximately half the pool of Smac/DIABLO to the cytosol. By contrast, transfection with the PERK siRNA promoted only modest cytosolic release of Smac/DIABLO and cytochrome c despite the fact that the death rate induced by extinction of PERK was comparable to that induced by STS. The Δ mito/cyto value for Smac/DIABLO (Figure 7B) was clearly positive for cells transfected with the PERK siRNA or with the non-targeting siRNA, indicating that under these conditions, Smac remained mostly mitochondrial. By contrast, the Δ mito/cyto value for Smac/DIABLO was close to zero for cells treated with STS indicating that Smac was equally distributed in the mitochondrial and cytosolic fractions. This difference was even more striking for cytochrome c since its Δ mito/cyto value was positive for cells transfected with the PERK siRNA but negative for STS-treated cells (Figure 7C). This indicates that the cytosolic form of cytochrome c largely predominates over its mitochondrial form in the latter but not in the former culture condition. Altogether these data demonstrate that PERK extinction, as opposed to a *bona fide* pro-apoptotic drug, causes only marginal cytosolic release of mitochondrial apoptogenic factors. Prior transfection of cells with the PERK siRNA but not with the non-targeting siRNA significantly reduced the staurosporine-induced cytosolic release of both Smac/DIABLO and cytochrome c as reflected by the increase of the Δ mito/cyto values in cells that received the PERK siRNA and the staurosporine treatment as compared with cells co-treated with the non-targeting siRNA and staurosporine (Figure 7, B and C). These data demonstrate that PERK extinction impairs the apoptogenic function of the mitochondria. We next checked whether this phenomenon had repercussions on formation of a functional apoptosome. For this purpose, fluorigenic substrates were used to measure activation of caspases-9 and 3 in lysates of NCI-H929 cells that were treated with staurosporine or with the PERK siRNA used either alone or in combination. Neither the PERK nor the non-targeting siRNA induced activation of caspase-9 and caspase-3 (Figures 7D and E) while staurosporine promoted activation of both caspases. Prior transfection of NCI-H929 cells with the PERK siRNA but not with the non- targeting siRNA prevented activation of caspases-9 and -3 in response to staurosporine. Altogether our data suggest that PERK extinction impairs the apoptogenic function of the mitochondria and causes an apoptosome deficiency that prevents activation of the executioner caspase-3.

## Discussion

Here we show that knocking-down a single UPR sensor promotes the autophagic death of human MM cell lines and the concomittant repression of apoptosis. The question as to whether autophagy is a death execution pathway is still a debated issue. Certain authors prefer to substitute the concept of death accompanied by autophagy to that of death executed by autophagy [24], [25]. It has been reported for example that autophagy can be required for initiation of conventional death effector mechanisms such as apoptosis or necrosis. Salazar et al. have recently shown that the cell death of human glioma cells induced by cannabinoids is preceded by activation of the UPR and is accompanied by massive autophagy [28]. Although cell death in this model is clearly executed by apoptosis, it is repressed by pharmacological and genetic inhibition of the autophagic machinery. Other authors defend the position that autophagy is also a legitimate means of self-killing (Eisenberg-Lerner et al. Cell Death And Differentiation, 2009). In support of the latter view, several recent studies have convincingly demonstrated that prolonged or exagerated autophagy can culminate in cell death [29], [30]. We show here that two pharmacological inhibitors that interfere either with the early or late stage of autophagy, similarly repress the death of MM cells induced by PERK knock-down. These results suggest either that autophagy is the death execution mechanism or that it cooperates with necrosis to cause cellular destruction. Our results do not allow us to discriminate between these two hypotheses but they do support the notion that apoptosis is not the death mechanism at play in our model.

Several papers have established that an excessive accumulation of ER stress activates the cytotoxic function of the UPR. For caspase 3/7 deficient MEFs cells exposed to radiation [31] as well as for cancer cells [32] exposed to the chemotherapeutic drug 4HPR, the ER stress-induced cytotoxicity is mediated via the PERK/eIF-2 alpha pathway. Other branches of the UPR can also promote cell death as exemplified by the fact that the susceptibility of PERK−/− insulin-producing ß cells to programmed cell death is linked to exacerbation of the IRE1 signaling pathway [13]. We have observed that a single UPR sensor knock-down induces a transient burst of UPR signaling via the other two intact stress sensors. This finding is compatible with the hypothesis that the transient silencing of a stress sensor in MM cells partially releases the control exerted by the UPR on Ig synthesis in the ER thereby maximizing ER stress. This induces in return a shift from a cytoprotective to a terminal UPR. This hypothesis is in agreement with recent studies conducted on MM cells showing that amplifying the intensity of the UPR by combining the proteasome inhibitor Bortezomib with a calcium channel blocker [33] or with a mimetic of phosphorylated eIF-2 alpha [34] tips the UPR balance from survival to death. However, while PERK−/− insulin-producing ß cells and MEFs undergo apoptosis as the result of PERK extinction [13], [14], PERK silencing in MM triggers their autophagic cell death. We have clearly excluded that the reasons for this discrepancy relate to the apoptosis-competence of MM cells since they underwent oligonucleosomal DNA fragmentation in response to staurosporine or to pharmacological ER stressors. The parameters that determine cell fate after UPR activation are not fully understood. According to Rutkowski et al., the UPR simultaneously activates the mediators that promote adaptation to stress (BiP) and those that induce apoptosis (CHOP) but the latter have a shorter half-life than the former [35]. A weak stress signal favours the clear-cut dominance of adaptation mediators and will promote cytoprotection. Conversely, when cells are exposed to a robust and persistent ER stress, protective mechanisms are overwhelmed by apoptotic mediators thereby committing cells to apoptosis. In other terms, the UPR commitment to survival or death is determined by the overall level of activation of the UPR sensors. We would like to propose that transient knock-down of PERK generates a weaker ER stress signal than that elicited by complete invalidation of the PERK gene and that the milder UPR that ensues is responsible for the choice of autophagic cell death in our model. In line with our present findings, Zismanov and colleagues have recently documented that overexpression of the tetraspanin CD81 or CD82 in human MM cell lines induces UPR signaling that is followed by non-apoptotic cell death that exhibits the hallmarks of excessive autophagy [36]. It would be worthwhile examining whether cell death promoted by extinction of stress sensors is restricted to protein-secreting cells and whether the choice of autophagic cell death in response to heigthened ER stress is related to the propensity of such cells for increased basal autophagic activity.

The connection between autophagy and ER stress has been established both for yeast [37] and mammalian cells [38], [39], [40]. In our experimental model, we bring evidence that the autophagic program also contributes to block the apoptosis pathway in MM cells. This assertion is substantiated by two sets of data. First, the PERK siRNA inhibited DNA fragmentation triggered by staurosporine. Second, this inhibition was partially reversed by the autophagy inhibitor 3-MA. Repression of apoptosis by the autophagic pathway can thus be envisaged as an element of the global survival strategy set up by moderate ER stress.

Assuming that UPR simultaneously activates both survival and apoptosis mediators, the fate of stressed cells is fairly predictable at both extremes of the ER stress spectrum. However, there may be a range of stress signals of intermediate intensity for which the balance between protective and cytotoxic pathways does not allow a clear-cut binary decision between survival and death. The ER stress generated by knock-down of a single UPR sensor might fall into this category. The moderate liberation of mitochondrial apoptogenic mediators observed in our experimental setting suggests that an apoptotic program is indeed launched by PERK knock-down but is compromised by the concomitant onset of the autophagic program. These mitochondrial damages would nevertheless be sufficient to be perceived by the cells as irreparable thus prompting execution of the autophagic death pathway by default. The incomplete loss of mitochondrial outer membrane permeability caused by PERK knock-down and the aborted apoptosis program that follows could be instrumental in promoting the switch from cytoprotective to cytotoxic autophagy. The alternate possibility is that PERK knock-down induces a cytoprotective UPR that is translated into a survival response that also exploits autophagy to repress apoptosis. The ER occupies a very large volume in MM cells and dilated ER vesicles in stressed MM cells are likely to be prominent target of the autophagic process. Under such conditions, autophagy could be induced to such high levels that MM cells virtually eat themselves to death.

Suppression of the apoptotic program by autophagy has been reported in other systems [41], [42], [43] but the novel notion that emerges from our findings is that autophagy represses apoptosis by impairing the apoptogenic function of the mitochondria. Our data suggest that certain components of the autophagic pathway can lock mitochondria in an “apoptosis-refractory state” characterized by their reduced ability to release key apoptogenic factors in response to an apoptosis-inducing stimulus. It is noteworthy that PERK silencing by itself induces some relocation of Smac and cytochrome c in the cytosol. This could possibly reflect an aborted attempt of some mitochondria to launch an apoptotic program. In spite of this, we found no evidence for caspase-9 nor caspase-3 activation in cells transfected with the PERK-targeting siRNA. This observation is coherent with the hypothesis proposed by Waterhouse et al [44] that a threshold of production of apoptogenic factors may be required for apoptosome formation and subsequent triggering of the downstream apoptotic machinery. It has also been reported that the cytoplasmic accumulation of cytochrome c is insufficient for caspase activation in neurons [45]. It is thus possible that the concomitantly reduced production of apoptogenic co-factors such as Smac/DIABLO contributes to limit the apoptosis-promoting function of cytochrome c in MM cells transfected with the PERK siRNA. Altogether these observations tend to suggest that the alterations of the mitochondrial apoptogenic function induced by the autophagic machinery are quantitative rather than qualitative. It had been documented that the autophagic process represses apoptosis by regulating the level of caspase activity [43]. Our present data suggest that the control of caspase activation by components of the autophagic pathway is indirect and operates through repression of the production of death effectors by the mitochondria thus preventing formation of a functional apoptosome.

Proteasome inhibitors have recently been shown to efficiently kill myeloma cells through induction of a terminal UPR [46], [47], [48] and excellent responses were obtained with first line proteasome inhibitors in this disease. This observation and our present finding that extinction of the UPR sensors promotes autophagic cell death of human MM cell lines give credit to the idea that targeting the UPR pathway is a promising therapeutic approach.

## Materials and Methods

### Multiple Myeloma-derived cell lines

Two human myeloma cell lines were used: U266 was purchased from the American Type Culture Collection (Manassas, VA) and NCI-H929 was obtained from Dr. B. Sola (Université de Caen, Basse-Normandie, France). All cells were cultured in RPMI 1640 supplemented with 10% SVF, 1 mM L-glutamine, 100 U/ml penicillin, 100 µM streptomycin and 2% HEPES (all from Invitrogen Life Technologies, Cergy-Pontoise, France).

### Reagents

The pan-caspase inhibitor zVAD-fmk was purchased from Bachem (Voisins-Le-Bretonneux, France). Staurosporine, tunicamycin, 3-methyladenine (3-MA) and Bafilomycin A1 were purchased from Sigma-Aldrich (St-Quentin Fallavier, France).

### RNA interference

Transfection was conducted with the TransIT-TKO Transfection Reagent (Mirus, Madison USA) according to recommandations of the manufacturer. The PERK, ATF6 and IRE1 siRNAs were synthesized by Ambion. Their targeting sequences were as follows: PERK (NM_004836, EIF2AK3, targeting sequence: sense GCAUGCAGUCUCAGACCCAtt and antisense UGGGUCUGAGACUGCAUGCtt); ATF6 (NM_007348), targeting sequence: sense CACAACAGAGUCUCAGGtt and antisense CCUGAGAGACUCUGUUGUGtt); IRE1 (NM_001433, ERN1, targeting sequence: sense GAUGUCCCACUUUGUGUCCtt and antisense GGACACAAAGUGGGACAUCtt). A non-targeting siRNA (Silencer Negative Control # 1) also purchased from Ambion was used as negative control in our experiments.

### Detection of cell death

Membrane alterations were revealed by quantification of phosphatidylserine (PS) exposure using FITC-conjugated annexin V (Bender MedSystems, Vienna, Austria) and by propidium iodide (PI) staining. Viable cells with intact membranes exclude PI, whereas the membranes of dead and damaged cells are permeable to PI. Annexin V is a 35–36 kDa Ca2+ dependent phospholipid-binding protein that has a high affinity for PS, and binds to cells with exposed PS. Annexin V may be conjugated to fluorochromes including FITC. In apoptotic cells, the membrane PS is translocated from the inner to the outer leaflet of the plasma membrane, thereby exposing PS to the external cellular environment. Since externalization of PS occurs in the earlier stages of apoptosis, FITC Annexin V staining can identify apoptosis at an earlier stage. Disruption of the mitochondrial transmembrane potential (ΔΨm) was revealed using the tetramethylrhodamine ethylester perchlorate (TMRE, Invitrogen, France). DNA fragmentation was assessed either by TUNEL assay (terminal deoxyribonucleotide transferase-mediated dUTP-X-nick-end labeling) using the In Situ Cell Death Detection Kit (Roche Applied Science, Manheim, Germany) or using the F 7–26 mAb from Alexis (Apostain, Laufelfingen, Switzerland) that detects DNA single strand breaks.

### Antibodies for Western blotting

The anti-human PERK, ATF6 alpha and XBP-1 polyclonal Abs were purchased from Santa Cruz Biotechnology (Santa Cruz, CA). The anti-caspase-3 and anti-cytochrome c Abs were purchased from BD Biosciences (BD Pharmingen, San Diego, CA). The anti-IRE1α polyclonal Ab and the anti-caspase-4 mAb were purchased from Novus Biologicals (Littleton, CO) and MBL (Woburn, MA) respectively. The anti-β Actin mAb was purchased from Sigma-Aldrich (St Quentin Fallavier, France). The anti-cytochrome oxidase mAb was purchased from Molecular Probes (Invitrogen Corp, CA) and the polyclonal anti Smac/DIABLO Ab was purchased from Pro-Sci. Inc (Poway, CA). The anti-LC3 mAb was purchased from MBL (Naka-ku Nagoya, Japan). The HRP-conjugated Abs used for revelation were purchased from Amersham (BD Biosciences, Little Chalfont, UK). The anti EiF-2 alpha monoclonal Ab was purchased from Biosource (Biosource Europe, Nivelles, Belgium) and the anti phospho EiF-2α was purchased by Cell signaling (Beverley, MA).

### Subcellular fractionation

Cytosolic and mitochondrial fractions were obtained using a digitonin-based subcellular fractionation technique as previously described [49].

### RNA isolation and quantitative real-time RT-PCR

Total RNAs were isolated with Trizol reagent (Invitrogen Life Technologies) according to the manufacturer's protocol. cDNAs were synthesized by extension of a mix of oligo (dT) and random primers with Super Script II reverse transcriptase (all from Invitrogen Life Technologies) and used for quantitative PCR analysis as previously described [50]. The primer sequences (forward/reverse) used were as follows: **PERK**, 5′-CGAAGTGGATGGAGATGATGATGC-3′/3′-AGTCTAGGATTTACAGCCAGGAAGC-5′, **ATF6**, 5′-TCCTCCACCTCCTTGTCAGC-3′/3′-TCATTCTTCTTGACTTGGTCCTTTC-5′, **IRE1**, 5′-TTGATAGAGAAGAT GATTGCGATGG-3′/3′-CCATCCAGGGATTCCTTTTCTATTC-5′, **GAPDH**, 5′-TGAAGGTCGGAGTCAACGGATTTG-3′/3′-CATGTGGGCCATGAGGTCCACCAC-5′.

The relative quantity of each transcript was normalized according to the expression of the housekeeping gene GAPDH.

### Transmission electron microscopy

Culture cells were fixed with 2% glutaraldehyde in culture medium (15 min) and in 2% glutaraldehyde-0.1 M NaCacodylate/HCL, pH 7.4 (30 min), washed three times in 0.2 M NaCacodylate/HCL, pH 7.4 and then post fixed with 1% OsO_4_-0.15 M NaCacodylate/HCL, pH 7.4 (30 min). After dehydration in a growing gradient of ethanol (30, 50, 70 and 95%, 5 min for each step), impregnation steps and inclusion were performed in Epon and finally polymerized at 60°C for 48 h. The sections were cut, contrasted with uranyl acetate and lead citrate before analysis with a JEOL 100CS electron microscope.

### Labelling of autophagic vacuoles with monodansylcadaverine

Cells were incubated with 0.05 mM monodansylcadaverine (MDC) [51] (Sigma Aldrich) at 37°C for 10 min and analyzed either by flow cytometry or by fluorescence microscopy.

### Measurement of caspase-3 and -9 activities

Caspase-3 and caspase-9 activities were estimated with DEVD-AMC and LEHD-AMC respectively, according to instructions of the manufacturer (Biomol International LP, TEBU BIO, France).

### Statistical analysis

The analysis was based on non-parametrical methods. We used a two-tailored *t*-test to analyze the significance of the differences observed in distinct experimental conditions as indicated in the figures. (* *p*<0.05; ** *p*<0.01; ***p<0.005)
